# Neural Correlates of Rewarded Response Inhibition in Youth at Risk for Problematic Alcohol Use

**DOI:** 10.3389/fnbeh.2017.00205

**Published:** 2017-11-03

**Authors:** Brenden Tervo-Clemmens, Alina Quach, Beatriz Luna, William Foran, Tammy Chung, Michael D. De Bellis, Duncan B. Clark

**Affiliations:** ^1^Department of Psychology, University of Pittsburgh, Pittsburgh, PA, United States; ^2^Center for the Neural Basis of Cognition, University of Pittsburgh, Pittsburgh, PA, United States; ^3^Department of Psychiatry, University of Pittsburgh, Pittsburgh, PA, United States; ^4^Department of Psychiatry and Behavioral Sciences, Duke University Medical Center, Durham, NC, United States

**Keywords:** adolescence, response inhibition, reward, substance use, risk factors, functional magnetic resonance imaging (fMRI)

## Abstract

Risk for substance use disorder (SUD) is associated with poor response inhibition and heightened reward sensitivity. During adolescence, incentives improve performance on response inhibition tasks and increase recruitment of cortical control areas (Geier et al., 2010) associated with SUD (Chung et al., 2011). However, it is unknown whether incentives moderate the relationship between response inhibition and trait-level psychopathology and personality features of substance use risk. We examined these associations in the current project using a rewarded antisaccade (AS) task (Geier et al., 2010) in youth at risk for substance use. Participants were 116 adolescents and young adults (ages 12–21) from the University of Pittsburgh site of the National Consortium on Adolescent Neurodevelopment and Alcohol [NCANDA] study, with neuroimaging data collected at baseline and 1 year follow up visits. Building upon previous work using this task in normative developmental samples (Geier et al., 2010) and adolescents with SUD (Chung et al., 2011), we examined both trial-wise BOLD responses and those associated with individual task-epochs (cue presentation, response preparation, and response) and associated them with multiple substance use risk factors (externalizing and internalizing psychopathology, family history of substance use, and trait impulsivity). Results showed that externalizing psychopathology and high levels of trait impulsivity (positive urgency, SUPPS-P) were associated with general decreases in antisaccade performance. Accompanying this main effect of poor performance, positive urgency was associated with reduced recruitment of the frontal eye fields (FEF) and inferior frontal gyrus (IFG) in both a priori regions of interest and at the voxelwise level. Consistent with previous work, monetary incentive improved antisaccade behavioral performance and was associated with increased activation in the striatum and cortical control areas. However, incentives did not moderate the association between response inhibition behavioral performance and any trait-level psychopathology and personality factor of substance use risk. Reward interactions were observed for BOLD responses at the task-epoch level, however, they were inconsistent across substance use risk types. The results from this study may suggest poor response inhibition and heightened reward sensitivity are not overlapping neurocognitive features of substance use risk. Alternatively, more subtle, common longitudinal processes might jointly explain reward sensitivity and response inhibition deficits in substance use risk.

## Introduction

Poor response inhibition and heightened reward sensitivity have been suggested as neurobiological risk factors for problematic substance use (Heitzeg et al., 2015). During adolescence, functional brain development supports the integration of these processes, which may influence risk for substance use initiation (Heitzeg et al., 2015; Luna and Wright, 2016). However, it is unclear how brain processes supporting cognitive-reward integration may incur risk for substance use disorders and whether these are associated with trait-level psychopathology and personality features of substance use risk.

Neuroimaging research in adolescents suggests substance use risk is associated with reduced BOLD activation in cortical brain regions supporting cognitive control. During response inhibition tasks, reduced prefrontal BOLD activation has been observed in youth who would later transition to heavy alcohol use (Norman et al., 2011) (Mahmood et al., 2013) and adolescents with a family history of alcoholism (Schweinsburg et al., 2004; see Heitzeg et al., 2015 for review). Response inhibition continues to improve through adolescence across a range of tasks (e.g., antisaccade; see Luna et al., 2010 for review), accompanied by functional changes in cortical control regions (Ordaz et al., 2013). Accordingly, poor response inhibition (c.f., Nigg et al., 2006) and reduced prefrontal BOLD activation (Norman et al., 2011) during adolescence may serve as risk factors for escalation to problematic substance use later in development.

Reduced engagement of cognitive control circuitry in youth at risk for substance use may underlie common behavioral associations between laboratory measures of response inhibition and trait-level psychopathology features of substance use risk (c.f., Young et al., 2009). To this end, neurobehavioral disinhibition (ND), a latent construct designed to asses an individual's general level of inhibition across affective, personality, and cognitive domains (c.f., Tarter et al., 2003), predicts substance use initiation (Tarter et al., 2003) and has been associated with reductions in BOLD activation in frontal cortex (McNamee et al., 2008).

Although poorer response inhibition and reduced prefrontal function have received support as neurocognitive indicators of adolescent substance use risk, previous work has typically investigated these associations in the absence of incentives on performance. However, a number of studies have demonstrated that adolescents' performance on response inhibition tasks improves when working toward an incentive (Jazbec et al., 2006; Hardin et al., 2007; Geier and Luna, 2012). Furthermore, rewarded response inhibition tasks increase BOLD activation in cortical control regions in adolescents with substance use disorder (SUD; Chung et al., 2011). This enhanced BOLD activation in youth with SUD in the context of reward may occur through enhanced reward signals from the ventral striatum, a brain region involved in the salience of reward cues (Berridge, 2007). Previous work suggests compulsive drug use is associated with a sensitization of ventral-striatal reward pathways (Robinson and Berridge, 1993). Alternatively, reward sensitivity may precede substance use initiation, as trait-level psychopathology features of substance use risk (externalizing symptoms: Bjork et al., 2010a; impulsivity: see Plichta and Scheres, 2014 review) are associated with increased BOLD activation in the ventral striatum.

In the current project, we utilized a rewarded antisaccade (AS) task to examine whether incentives enhanced response inhibition in youth at risk for substance use in a large adolescent neuroimaging sample with two time points. Building on previous work using this rewarded AS task in a normative developmental sample (Geier et al., 2010) and adolescents with SUD (Chung et al., 2011, 2015), we hypothesized that incentives would improve performance and reduce the association between substance use risk and poorer response inhibition. We first examined the relative prediction of multiple substance use risk factors on AS performance and BOLD activation. Based on the neurobehavioral disinhibition model, we predicted substance use risk factors representing poor impulse control (e.g., trait impulsivity and externalizing symptoms) would be associated with poorer AS performance and lower BOLD activation in cortical control areas. We further hypothesized these differences would be moderated by incentives, with at-risk youth showing greater reward-related improvement in response inhibition and smaller differences in inhibition-related BOLD activation during reward, relative to neutral conditions.

## Methods

### Participants

Participants were recruited at the University of Pittsburgh site of the National Consortium on Adolescent Neurodevelopment and Alcohol (NCANDA) study, using procedures detailed in Brown et al. (2015). In brief, targeted catchment area calling was used to initially identify eligible youth (e.g., ages 12–21). In order to study prospective prediction for substance use risk, NCANDA recruited youth with limited substance use. However, sampling also prioritized adolescents at increased risk for alcohol use disorder (AUD) (e.g., family history of SUD), such that youth with increased risk represented 47% of the total NCANDA sample. Exclusion criteria included MRI contraindications (e.g., claustrophobia, pregnancy, non-removable metal in the body), medical history that may influence MRI (e.g., head injury with loss of consciousness), current or persistent psychiatric disorder that may influence study completion (e.g., psychosis), and psychiatric medication (see Brown et al., 2015). The University of Pittsburgh's Institutional Review Board approved the study. Adult participants provided informed consent. For minors, parents provided informed consent and youth provided assent. Participants were compensated for completing research assessments.

Based on study-specific exclusion criteria (see below), the current analyses included 116 participants who provided useable data at baseline and/or 1-year follow up. This final analysis sample spanned adolescence into young adulthood (baseline age: 12.27–21.96, mean = 17.10, *SD* = 2.60); 56.90% (*n* = 66) female; and represented 80.17% White (*n* = 93), 18.10% (*n* = 21) Black, and 1.72 % (*n* = 2) multi-racial or other race/ethnicity (see Table 1 for sample description). A majority (58%) of participants contributed data at both baseline and follow up (total sessions: *N* = 183; total subjects: *N* = 116; subjects at baseline: *n* = 101; subjects at follow up: *n* = 82; subjects with data at both visits: *n* = 67). As detailed below, we utilize methods robust to missing data (linear and generalized mixed effects models) and therefore include all available data (subject at visit).

**Table 1:** Subject characteristics in risk categories.

	**EXT**		**INT**		**FH**		**ETD**		**Any Risk (Non-ETD)**	
	**Risk**	**No risk**	**Risk**	**No risk**	**Risk**	**No risk**	**ETD**	**Non-ETD**	**Any risk**	**No risk**
Number of Participants	31	85	26	90	21	95	31	85	40	45
Gender n female	19	47	18	48	9	57	19	47	21	26
Age (years)	17.35(2.55)	16.98(2.64)	17.72(2.25)	16.89(2.69)	17.38(2.85)	17.01(2.57)	19.20(1.47)	16.30(2.51)	16.30(2.34)	16.30(2.67)
Socioeconomic status (standard score)	81.13(15.97)	92.73(12.31)	88.31(15.83)	89.82(13.95)	84.80(15.50)	90.50(13.97)	89.28(15.75)	89.53(13.93)	84.88(14.74)	94.07(11.55)
Generalized ability (z-score)	−0.389(0.967)	0.085(0.796)	0.191(0.795)	−0.105(0.880)	0.038(0.820)	−0.055(0.880)	0.199(0.839)	−0.112(0.865)	−0.205(0.938)	−0.049(0.800)
Positive urgency	1.96(0.612)	1.77(0.608)	1.85(0.629)	1.82(0.611)	1.89(0.671)	1.81(0.602)	1.85(0.756)	1.82(0.557)	1.96(0.562)	1.69(0.526)
Negative urgency	2.10(0.621)	1.85(0.641)	2.03(0.609)	1.88(0.652)	1.82(0.520)	1.93(0.667)	1.98(0.699)	1.89(0.628)	2.06(0.559)	1.74(0.657)

*All scores from baseline visit. In addition to EXT, INT, and FH, Any Risk (Non−ETD) additionally excludes EOS (n = 9). See Table 2 for associations between variables*.

### Measures

#### Exceeds threshold drinking

At baseline, based on self-report from the Customary Drinking and Drug Use Record (CCDR) (Brown et al., 1998), 31 participants in the analysis sample reported alcohol use that exceeded age-adjusted National Institute on Alcohol Abuse and Alcoholism (NIAAA) guidelines for risky drinking (see Brown et al., 2015), and are considered “exceeds threshold drinkers” (ETD).

#### Risk factors for substance use

As with other sites, the NCANDA Pittsburgh sample was recruited such that approximately half of the participants were at increased risk for problematic alcohol use. The current sample utilizes participants with increased substance use risk based on one or more of the following: early substance use onset (EOS; *n* = 9), family history of substance use disorder (FH; *n* = 21), externalizing symptoms (EXT, *n* = 31), or internalizing symptoms (INT, *n* = 26). In the current analyses, EOS, FH, EXT, and INT were categorical variables (Risk vs. No Risk) and were defined at the baseline visit as in Brown et al. (2015).

EOS was defined as consuming the first full drink of alcohol prior to age 15, based on the child's report on the CDDR (Brown et al., 1998). FH was defined as having at least one biological parent with a history suggesting a substance use disorder (SUD), based on parent report using the Family History Assessment Module (Rice et al., 1995). EXT was defined as having endorsed at least one symptom of conduct disorder or antisocial personality disorder on the Computerized Semi-Structured Assessment for the Genetics of Alcoholism (SSAGA, Bucholz et al., 1994; Hesselbrock et al., 1999) or having an Achenbach System of Empirically Based Assessment (ASEBA; Achenbach, 2009) externalizing age- and gender-adjusted t-score above 60. Similarly, INT was defined as having endorsed two or more symptoms or having an ASEBA internalizing t-score above 60. In the current analysis, we did not examine EOS because of the few participants meeting criteria in the Pittsburgh sample (*n* = 9).

#### Trait impulsivity

In addition to risk factors described by Brown et al. (2015), we also examined associations between AS performance and BOLD activation and trait impulsivity as measured by the short version of the UPPS-P (SUPPS-P, Cyders et al., 2014). Trait impulsivity is associated with increased vulnerability to SUD (c.f., Verdejo-García et al., 2008) and has been conceptualized as having shared underlying features with antisocial behavior and substance use (Krueger et al., 2007). Although trait impulsivity and response inhibition have both been considered measures of inhibitory control, it has been suggested trait personality measures of impulsivity likely represent more global differences in impulsive choice whereas behavioral measures of response inhibition reflect specific cognitive processes (Reynolds et al., 2006). We focused on the SUPPS-P scales of negative and positive *urgency* as the urgency domain has been associated with substance use (Zapolski et al., 2009) and BOLD activation in a rewarded stop signal task (Wilbertz et al., 2014). Participants completed the SUPPS-P at both baseline and follow up visits.

#### Generalized cognitive ability

As part of the NCANDA test protocol, participants completed the Penn Computerized Neurocognitive Battery (WebCNP: Gur et al., 2010) and traditional “pencil and paper” neuropsychological tests (Sullivan et al., 2016). We utilized a composite score, generalized ability accuracy (GA; see Sullivan et al., 2016), as an outcome variable and covariate when examining substance use risk factors in our analyses. The GA measure was derived from several domains of neuropsychological function, allowing us to examine whether associations among substance use risk factors, AS performance, and BOLD activation were specific to response inhibition or instead, reflected reduced generalized cognitive ability.

#### Socioeconomic status

The socioeconomic status (SES) variable was determined by both parental education and income (see Brown et al., 2015). SES was expressed as a standard score (mean = 100, *SD* = 15) and included as a covariate in secondary analyses.

#### Baseline and follow up coding of primary variables of interest

In order to harmonize our analytic approach with previous work with NCDANDA baseline data (c.f., Brown et al., 2015; Müller-Oehring et al., 2017), at both baseline and follow up we utilized scores from study initiation for the primary substance use risk categories (FH, EXT, INT) and exceeds threshold drinking (ETD) as outlined in Brown et al. (2015). This approach allows our results to be interpreted more readily with other projects describing cognitive differences with baseline risk group definitions (c.f., Sullivan et al., 2016). Continuous primary measures (participant age and trait-impulsivity) were treated as time-varying covariates, with the unique scores entered at baseline and follow up.

#### Missing data from primary variables of interest

Based on the random effects structure of our modeling framework (see below), we were able to utilize participants that only had data at one visit (baseline or follow up). However, some participants were missing primary variables of interest. In the final behavioral sample (see below), SUPPS-P data was missing for two participants at baseline and eight participants at follow up. In the final neuroimaging sample two participants were missing SUPPS-P at baseline. As SUPPS-P was collected at both baseline and follow up, missing sessions (subject at visit) were excluded from analyses of SUPPS-P variables. One subject included in both behavioral and neuroimaging analysis was missing GA data. Six participants were missing SES data. As GA and SES were defined at baseline these participants were excluded from all analyses using these variables. No participants were missing FH, EXT, INT, or ETD data.

### Rewarded antisaccade task

**Figure 1:**
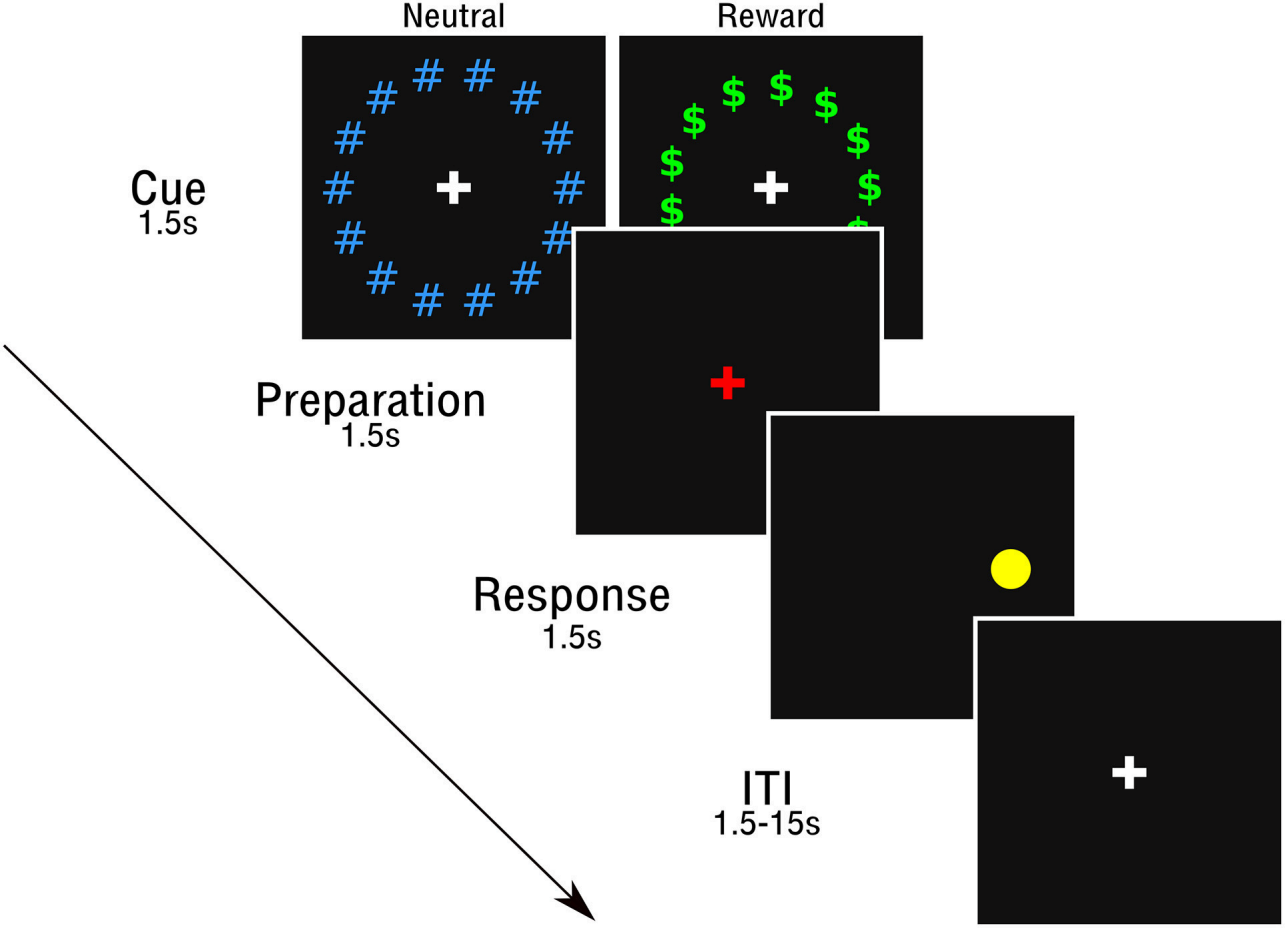
Rewarded antisaccade task.

Participants completed the same rewarded antisaccade task as used in Geier et al. (2010) and Chung et al. (2011) (See Figure 1). The full protocol included 56 full reward trials and 56 full neutral trials, completed across four neuroimaging runs (14 reward and 14 neutral trials per run), which included three epochs (cue, preparation, and response). Full trials began with a cue epoch (1.5 s), where participants viewed a white central fixation cross, surrounded by a circle of green “$” symbols (reward trials) or blue “#” symbols (neutral trials). Next, a red fixation cross appeared, indicating the preparation epoch (1.5 s), where subjects prepared to stop an impending eye movement to an unknown location. Finally, in the response epoch (1.5 s), a peripheral cue (yellow circle) was presented along the horizontal meridian at 1 of 6 eccentricities (±3, 6, and 9 degrees visual angle, relative to fixation) and participants were instructed to perform a saccade away from the circle toward the mirror location. An additional 24 (12 rewarded; 12 neutral) partial trials that presented either the cue alone (6 of each reward type) or the cue and prep epoch (6 of each reward type) but not the response epoch were included in order to estimate the hemodynamic response for each trial epoch (c.f., Ollinger et al., 2001).

Prior to the session, participants were informed they could earn a small monetary reward during trials with the “$.” As in Chung et al. (2011), participants were told they could win up to $10 for correct antisaccade performance in the session and that there would be no monetary loss for incorrect responses. However, participants were not told the value of a specific response in reward trials and feedback was not provided. This prevented participants from attempting to keep track of an ongoing reward tally. At the end of the session, all participants received the full $10 reward.

#### Eye movement measurement and scoring

Stimuli from the AS task were presented on a flat screen behind the MRI scanner with E-Prime software (Schneider et al., 2002) and made visible to the subject with a mirror mounted on the head coil. Eye-tracking was performed using a long-range optics eye-tracking system (Applied Science Laboratories, Bedford, MA) and eye-position was measured through corneal reflection. Video monitoring was performed to ensure task compliance. Prior to the test session, a 9-point calibration was performed for each subject.

Eye movements in the response epoch were a scored as a correct AS if the first eye movement during the response epoch had a velocity greater than or equal to 30°/s (Gitelman, 2002) and was made in the mirror location of the peripheral cue and extended at least 2.5° visual angle from central fixation. In contrast, eye movements were marked as an incorrect AS if the first saccade in the response epochs was made toward the peripheral cue and extended at least 2.5° visual angle from central fixation, but then later directed toward the correct location, suggesting task compliance. Trials in which no saccade occurred or if the eye-tracker lost fixation were excluded from all analyses. Scan sessions were excluded from all behavioral and fMRI analysis if the proportion of excluded trials was greater than 33%.

Within the final analysis sample (*N* = 116), the overall proportion of excluded trials was 11.4% at baseline and 12.1% at follow up. Utilizing linear mixed effects models (see below for methods), the proportion of excluded trials was positively associated with the trait impulsivity measure of negative urgency [*t* = 2.01, $\chi_{\left(1\right)}^{2}$ = 4.02, *p* = 0.045], suggesting that those with higher levels of negative urgency had more excluded trials. Those with a family history of substance use had a significantly lower proportion of excluded trials [*t* = −1.97, $\chi_{\left(1\right)}^{2}$ = 3.87, *p* = 0.049]. Age at visit, EXT, INT, ETD, and positive urgency were not associated with the proportion of excluded eye tracking trials (see Supplemental Table 1).

### fMRI data acquisition

Imaging data were collected using a 3.0-T Siemens Magnetom TIM Trio at the Magnetic Resonance Research Center at the University of Pittsburgh. Structural images used for functional registration and conversion to a standardized template were collected using a magnetization prepared rapid acquisition gradient-echo (MP-RAGE) pulse sequence with a 160 slices (1.2 × 0.938 × 0.938 mm). Functional data were collected using an echo-planar imaging (EPI) sequence with the following parameters: TR = 1.5 s, TE = 28 ms, Flip Angle = 73°, and 64 × 64 acquisition matrix with a field of view of 200 mm. Twenty-nine slices were collected in the axial plane with 3.125 × 3.125 by 3.200 mm anisotropic voxels.

### fMRI data preprocessing

Preprocessing of functional data followed standard procedures. This included slice timing correction, despiking (AFNI 3dDespike), motion correction (mcflirt; Jenkinson et al., 2002), brain extraction, non-linear registration of functional data to a standardized anatomical brain (3 mm MNI-152 template: 2009c), spatial smoothing with FWHM of 5 mm (SUSAN; Smith and Brady, 1997), high pass filtering at 80 volumes (0.00625 Hz), and scaling by 10,000 of the global median.

### Data analysis

#### Associations among participant characteristics

Associations among participant characteristics were analyzed for sample description and covariate selection (Table 2). In order to provide standardized associations across all variable types, Pearson (continuous-continuous), polyserial (continuous-categorical), and polychoric (categorical-categorical) correlation values (Fox, 2010) are reported (see Table 2). Significance testing utilized Pearson correlation for continuous-continuous associations, Welch's *t*-test for continuous-categorical associations and chi-square testing for categorical-categorical associations.

**Table 2:** Correlations among participant characteristics.

	**EXT**	**INT**	**FH**	**ETD**	**PUG**	**NUG**	**Gender**	**Age**	**SES**	**GA**
EXT		Polychoric	Polychoric	Polychoric	Polyserial	Polyserial	Polychoric	Polyserial	Polyserial	Polyserial
INT	0.467^**^		Polychoric	Polychoric	Polyserial	Polyserial	Polychoric	Polyserial	Polyserial	Polyserial
FH	0.133	0.032		Polychoric	Polyserial	Polyserial	Polychoric	Polyserial	Polyserial	Polyserial
ETD	0.056	−0.085	0.223		Polyserial	Polyserial	Polychoric	Polyserial	Polyserial	Polyserial
PUG	0.176	0.031	0.068	0.032		Pearson	Polyserial	Pearson	Pearson	Pearson
NUG	0.231	0.135	−0.103	0.075	0.686^**^		Polyserial	Pearson	Pearson	Pearson
Gender	0.091	0.238	−0.241	0.091	–0.282^*^	−0.095		Polyserial	Polyserial	Polyserial
Age	0.086	0.194	0.080	0.705^**^	–0.244^*^	−0.095	0.136		Pearson	Pearson
SES	–0.432^*^	−0.060	−0.210	−0.010	−0.162	−0.109	−0.014	−0.005		Pearson
GA	–0.306^*^	0.209	0.062	0.226	–0.296^*^	–0.272^*^	0.033	0.433^*^	0.411^*^	

* *p < 0.05*,

** *p < 0.01. Lower triangle displays correlations. Upper triangle displays correlation type. EXT, INT, FH, and ETD were coded with meeting criteria/risk as 1. Gender was coded with women as 1. See (Data Analysis for further discussion). Significant relationships are bolded*.

#### Antisaccade behavioral performance

Behavioral analysis was performed using R 3.1.2 (R Core Team, 2014). Mixed effects models (lme4 package; Bates et al., 2013) were used to examine associations between antisaccade (AS) correct response rates (accuracy) and latency and exceeds threshold drinking (ETD), substance use risk factors (FH, INT, EXT), and trait impulsivity (positive and negative urgency) and whether these associations were moderated by reward.

AS correct response rates were analyzed with generalized mixed-effects models with a logit link function as trial level data was binomially distributed (correct vs. incorrect). AS latencies were analyzed with linear mixed effects models and only included correct trials. Both linear and generalized-linear mixed-effects models used maximum likelihood estimation. Random intercepts were estimated for each subject. Reward condition (reward/neutral), visit (baseline/follow up), age at visit, exceeds threshold drinking (ETD), substance use risk factors (FH, EXT, INT), trait impulsivity measures (positive and negative urgency), generalized ability (GA), and socioeconomic status (SES) were included as fixed effects in three types of models. In the first type (Type A), ETD, FH, EXT, INT, and impulsivity measures were examined as predictors of AS performance in separate models. Second (Type B), ETD, FH, EXT, INT, and impulsivity measures were examined as joint predictors of AS performance in the same model. Third (Type C), GA and SES were included as additional covariates to the joint model. Reward, visit, and age at visit were included in all three model types. Secondary analysis included gender as a covariate in analysis of positive urgency (see below). Significance values for fixed effects were obtained through the car package in R (Fox et al., 2016; Wald chi-square test). Given the correlation between our variables of interest and our goal of defining sensitive and specific associations between substance use risk factors and AS performance, we highlight predictors that are significant across all three model types (A-C).

#### fMRI

Twelve sessions were excluded from all fMRI analyses due to poor EPI coverage across imaging runs (*n* = 4) or technical errors (*n* = 8). Accordingly, the final neuroimaging sample consisted of 171 scan sessions distributed across 111 participants (participants at baseline, *n* = 95; participants at follow up, *n* = 76; participants with data at both visits, *n* = 60). Subject-level fMRI analyses were performed with Analysis and Visualization of Functional Neuroimages (AFNI, Bethesda, MD) software. In order to estimate the BOLD response at the trial- and individual AS epoch-level (cue, preparation, response), two level 1 GLM analyses were run for each subject at each visit using AFNI's 3dDeconvolve tool.

#### GLM-1

Reward and neutral trial-level BOLD responses were estimated with a 4,500 ms boxcar convolved with a gamma function (AFNI's block 4) and scaled to have an amplitude of 1. Separate regressors were included for correct, incorrect, and dropped/poor eye-tracking AS trials. Partial trials were modeled in the same manner but with 1,500 ms (cue partial trial) or 3,000 ms (cue and preparation partial trial) boxcars. Six rigid-body head motion parameters and their derivatives and run-wise 0 through 3rd order polynomials were used as nuisance regressors. The current and preceding TR were censored if the Euclidean norm head motion distance surpassed 0.9 mm, based on suggestions for task-based neuroimaging outlined in Siegel et al. (2014). Parameter estimates were examined both as main effects (Task > Fixation) and in comparisons with reward type (Reward > Neutral).

#### GLM-2

As reward-related changes in BOLD activation have been shown to vary according to demand characteristics (c.f., Geier et al., 2010), we also estimated BOLD activation in individual task-epochs (cue, preparation, and response). Epoch-level BOLD responses were estimated in a second model with individual regressors for cue, preparation, and response epochs. Relevant partial trials were included as examples of the epoch in question to aid in epoch-specific HRF estimation. All three epochs (cue, preparation, and response) were modeled with a 1,500 ms boxcar convolved with a gamma function (AFNI's block 4). The same nuisance regressors and motion censoring were used as in GLM-1.

#### Regions of interest

We first examined associations of BOLD activation with ETD and substance use risk (FH, EXT, INT, impulsivity) in a priori regions of interest (ROIs) (Table 3). Regions were selected based on their association with the AS task in previous voxelwise analyses (Velanova et al., 2009; Geier et al., 2010), including the frontal and supplementary eye fields (FEF and SEF), pre supplementary motor area (pre-SMA), and executive function more generally (c.f., Wesley and Bickel, 2014), including posterior parietal cortices (PPC), dorsolateral prefrontal cortex (DLPFC), ventrolateral prefrontal cortex (VLPFC), inferior frontal gyrus (IFG) and dorsal anterior cingulate cortex (dACC). Additionally, given the focus on interactions with reward, we also selected regions of interest from the striatum, including the caudate, putamen, and nucleus accumbens (NAcc), which have previously been associated with reward effects and externalizing symptoms (Bjork et al., 2010b) and rewarded response inhibition tasks (Wilbertz et al., 2014).

**Table 3:** Regions of interest and BOLD characteristics.

	**MNI X, Y, Z**	**Radius (mm)**	**Voxels (*n*)**	**Sessions excluded (*n*)**	**Neutral (*t*)**	**Reward (*t*)**	**Reward > Neutral (*t*)**	**Reward by hemisphere (*t*)**
**SUBCORTICAL**								
Caudate		–		0	3.22^**^	5.56^**^	3.15^**^	−0.02
Left	−13.7, 13.5, 9.5		137					
Right	12.0, 13.1, 11.0		154					
Putamen		–		1	18.63^**^	20.02^**^	2.01^*^	<0.01
Left	−25.1, 6.8, 0.5		136					
Right	24.3, 7.0, 0.4	–	150					
NAcc		–		3	−2.28^*^	1.17	3.86^**^	0.75
Left	−9.2, 12.4, −6.9		14					
Right	8.5, 13.4, −6.5		14					
**CORTICAL**								
PPC				0	14.65^**^	16.23^**^	2.23^*^	−0.01
Left	−32, −48, 50	10	85					
Right	32, −54, 48	10	97					
FEF				0	20.18^**^	21.76^**^	2.45^*^	0.08
Left	−25.5, −1.5, 46	10	122					
Right	26.5, −1.5, 58	10	89					
SEF	0.0, −4.6, 62.0	7	46	0	9.37^**^	10.74^**^	2.02^*^	–
pre-SMA	0.0, 5.0, 52.1	7	38	0	10.48^**^	11.38^**^	1.24	–
dACC	0.0, 19.5, 40.5	10	156	0	11.43^**^	13.80^**^	2.38^*^	–
DLPFC				0	−0.53	2.04^*^	2.78^**^	−0.49
Left	−41.0, 19.0, 41.0	12	103					
Right	42.0, 18.0, 42.0	12	169					
VLPFC				0	6.71^**^	7.83^**^	1.36	0.03
Left	−46.5, 10.5, 24.0	10	80					
Right	49.5, 12.0 22.0	10	95					
IFG				1	7.99^**^	8.11^**^	0.13	0.17
Left	−40.0, 6.0, 0.0	12	220					
Right	40.0, 10, 2.0	12	230					

* *p < 0.05*,

** *p < 0.01*.

Cortical ROIs were taken from a previous longitudinal neuroimaging study utilizing an AS task in adolescents (Ordaz et al., 2013), where the full methods are provided. Briefly, central coordinates were identified using topic and term searches in Neurosynth (http://neurosynth.org/), a meta-analysis tool for functional neuroimaging. Minor corrections to coordinates were made to ensure final ROIs overlapped with canonical eye movement regions (c.f., Munoz and Everling, 2004). From each central coordinate, spheres were grown, with the radius determined based on anatomical size and to avoid overlap (see Table 3). Striatum ROIs were taken from the Harvard-Oxford Atlas distributed through FSL software (Jenkinson et al., 2012). All ROIs were eroded such that they only included voxels with a 50% or greater probability of being gray matter in the MNI-152 template.

Our final list of ROIs included several pairs of bilateral regions (Table 3). In order to reduce the number of comparisons and because our hypotheses were not hemisphere specific, our primary analyses utilized left and right ROI pairs in one model with hemisphere as a within-subject factor. No ROI pair had a significant reward by hemisphere interaction (Table 3).

#### Region of interest testing

Nonzero mean BOLD activation (parameter estimates from GLM-1 & 2; see above) from correct trials were extracted from ROIs for each subject at each visit. As in the AS behavioral analysis, linear mixed effects models were utilized to examine the association of BOLD activation with ETD and substance use risk factors (FH, EXT, INT, impulsivity) and whether these associations were moderated by reward. Random intercepts were estimated for each subject and fixed effects were included in three phases (Type A, B, C). Additionally, session-wise motion estimates (proportion of censored volumes due to head motion) were used as a covariate in all models. Secondary analysis of positive urgency included gender as a covariate, as positive urgency was higher in males than females (see below). Scan sessions (subject at a particular visit) were excluded from analysis of an ROI if it did not have at least 90% epi coverage of the ROI or ROI pair. This exclusion never resulted in more than three scan sessions being omitted. In order to maximize the precision of individual subject estimates, we first examined main effects of ETD and risk factors (FH, EXT, INT, impulsivity) and their interactions with reward on trial-wise BOLD responses (GLM-1). Subsequently, we examined whether reward effects varied by AS epoch (reward by epoch interaction) using individual BOLD estimates of cue, preparation, and response from GLM-2. In order to assist in the interpretation of the direction of effects with BOLD activation, we additionally examined the association between BOLD activation and antisaccade correct response rate (accuracy). In all cases, significance values across ROIs were corrected for multiple comparisons using the false-discovery rate (FDR; *q* < 0.05).

#### Voxelwise testing

To ensure the selection of ROIs did not bias our results we also performed voxelwise linear mixed effects analysis (3dLME; Chen et al., 2013) with age at visit and session-wise motion as covariates (model Type A). As in ROI analysis, voxelwise analysis only used data from correct trials. Voxelwise testing was limited to voxels that met each of the following criteria: 50% or greater probability of being gray matter in the MNI-152 template, full EPI coverage in all participants across all runs, and a significant simple effect of the task in reward or neutral trials (task vs. fixation), suggesting activation to the AS task significantly differed (positively or negatively). When examining ETD and risk factors (FH, INT, EXT, impulsivity), multiple comparison correction within this voxelwise space was performed using the intersection of voxelwise FDR correction (*q* < 0.05) and cluster size. AFNI's 3dClustsim program was used to determine cluster size threshold through a Monte Carlo simulation with parameters derived from mean spatial autocorrelation parameters from GLM-1 residuals.

## Results

### Associations among participant characteristics

Based on the significant association of GA with EXT, PUG, and NUG and SES with EXT (Table 2), these variables were used as covariates in the step-wise modeling procedure (Type A-C). Additionally given that female participants had lower levels of positive urgency (mean female positive urgency baseline: 1.70; mean male positive urgency baseline: 1.98; polyserial correlation: −0.282, welch's *t*: −2.49, *p* = 0.014), secondary analysis of positive urgency included gender as a covariate.

### Antisaccade behavioral performance

#### Accuracy

**Figure 2:**
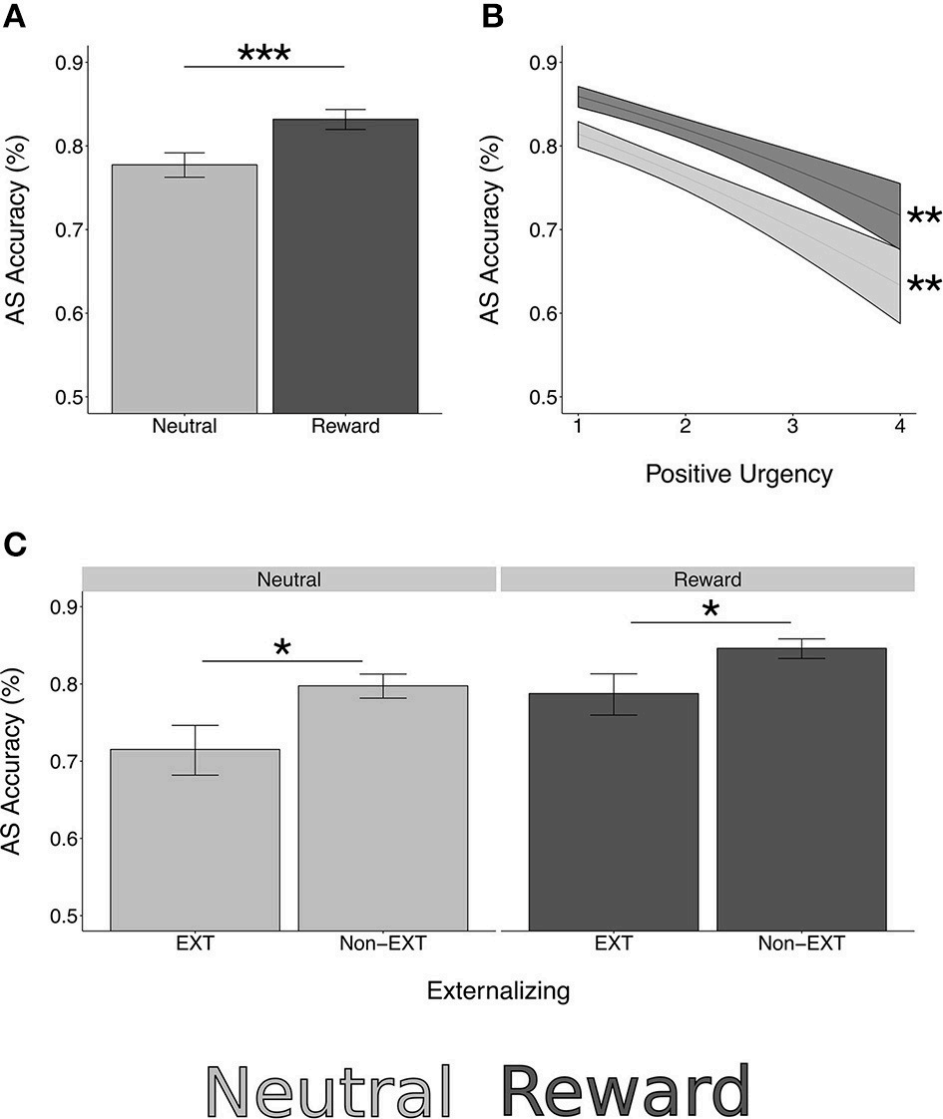
Rewarded antisaccade behavioral performance. ^*^p < 0.05, ^**^p < 0.01, ^***^p < 0.0001. **(A)** Antisaccade correct response rate (accuracy) is significantly higher in reward trials compared to neutral [*z* = 9.01, $\chi_{\left(1\right)}^{2}$ = 81.16, *p* < 0.0001]. **(B)** Positive urgency is a significant negative predictor of AS accuracy [*z* = −4.19, $\chi_{\left(1\right)}^{2}$ = 17.53, *p* < 0.001]. This association is not moderated by reward [*z* = 0.32, $\chi_{\left(1\right)}^{2}$ = 0.12, *p* = 0.750]. **(C)** Those with externalizing risk have lower AS accuracy [*z* = −2.34, $\chi_{\left(1\right)}^{2}$ = 5.50, *p* = 0.019]. This does not differ by reward type [*z* = 0.66, $\chi_{\left(1\right)}^{2}$ = 0.43, *p* = 0.510]. Simple effects testing confirmed significant effects of externalizing risk and positive urgency in both reward and neutral trials (externalizing neutral: *z* = −2.43, *p* = 0.015; externalizing reward: *z* = −2.10, *p* = 0.036; positive urgency neutral: *z* = −4.06, *p* = 0.001; positive urgency reward: *z* = −3.73, *p* = 0.002).

Overall, antisaccade (AS) correct response rate (accuracy) (mean = 75.31%, *SD* = 16.67%) was comparable with previous work (Geier et al., 2010). Consistent with previous work (Geier et al., 2010), participant age was a significant positive predictor of AS accuracy [*z* = 5.36, $\chi_{\left(1\right)}^{2}$ = 28.70, *p* < 0.001], with increased performance in older participants. Furthermore, as in Geier et al. (2010), AS accuracy was significantly higher in reward trials compared to neutral [*z* = 9.01, $\chi_{\left(1\right)}^{2}=81.16$, *p* < 0.001; Figure 2].

#### Substance use and risk factors

Externalizing risk [*z* = −2.34, $\chi_{\left(1\right)}^{2}=5.50$, *p* = 0.019] and higher levels of the trait impulsivity measure of positive urgency [*z* = −4.19, $\chi_{\left(1\right)}^{2}$ = 17.53, *p* < 0.001] were associated with lower AS accuracy while controlling for age and reward condition (model type A; Figure 2). Externalizing risk and positive urgency remained significant predictors when ETD and the other substance use risk factors were entered into a multivariate model (model type B; Table 4), suggesting that EXT and positive urgency were each independently associated with AS correct response rate controlling for other variables. INT, FH, ETD, and negative urgency were not significantly associated with AS accuracy (see Table 4).

**Table 4:** Main effects and reward interactions from models of antisaccade performance.

	**EXT**	**INT**	**FH**	**ETD**	**PUG**	**NUG**	**Age**	**SES**	**GA**
**ACCURACY (z)**									
Main effect	−2.34^*^^B^	1.12	−0.62	1.33	−4.19^**^^B^^,^^C^^,^^G^	−1.39	5.36^**^^B^^,^^C^	0.82	2.93^**^
Reward interaction	0.66	0.58	−0.63	−0.24	0.32	0.66	−1.56	0.42	−0.09
**LATENCY (t)**									
Main effect	0.04	0.32	−1.14	0.07	−2.80^**^^B^^,^^C^^,^^G^	−0.26	−3.32^**^^B^^,^^C^	0.55	0.25
Reward interaction	0.81	0.56	−1.30	0.45	0.42	0.69	−0.89	1.01	0.11

* *p < 0.05*,

** *p < 0.01. Displayed estimates are test statistics from models with the specific factor, subject age, visit, and reward condition (Type A)*.

B p < 0.05 (Type B) model with all risk factors, subject age, visit, and reward condition;

C *p < 0.05 (Type C) model with all risk factors, subject age, visit, reward condition, and socioeconomic status (SES) and GA (generalized ability). Significant estimates are bolded*.

G *Positive Urgency p < 0.05 while covarying gender*.

Neither externalizing risk nor positive urgency had significant interaction terms with the AS reward condition [externalizing risk by reward: *z* = 0.66, $\chi_{\left(1\right)}^{2}$ = 0.43, *p* = 0.510; positive urgency by reward: *z* = 0.32, $\chi_{\left(1\right)}^{2}$ = 0.12, *p* = 0.750], suggesting equivalent performance effects in reward and neutral trials (Table 4, Figure 2). Supporting this, simple effects testing revealed externalizing risk and positive urgency were significant predictors of AS accuracy in both neutral and reward trials [externalizing neutral: *z* = −2.43, *p* = 0.015; externalizing reward: *z* = −2.10, *p* = 0.036; positive urgency neutral: −4.06, *p* < 0.001; positive urgency reward: *z* = −3.73, *p* < 0.001]. Reward interactions were also not significant for INT, FH, ETD, or negative urgency (Table 4).

#### Covariate relationships

SES was not associated with AS accuracy [*z* = 0.82, $\chi_{\left(1\right)}^{2}$ = 0.68, *p* = 0.410]. Generalized cognitive ability (GA) was positively associated with AS accuracy [*z* = 2.93, $\chi_{\left(1\right)}^{2}$ = 8.57, *p* = 0.003]. When covarying GA (i.e., model type A + GA), the association between externalizing risk and AS accuracy was reduced to a trend [*z* = −1.69, $\chi_{\left(1\right)}^{2}$ = 2.84, *p* = 0.092], but positive urgency remained a significant negative predictor of AS accuracy [*z* = −3.88, $\chi_{\left(1\right)}^{2}$ = 15.09, *p* < 0.001].These results were unchanged in the full model with all other predictors (model Type C; see Table 4). Secondary analysis further showed positive urgency remained a significant negative predictor of AS accuracy while covarying gender [type A + gender: *z* = −4.30, $\chi_{\left(1\right)}^{2}$ = 18.52, *p* < 0.001]. Gender was not a significant predictor in this model [*z* = 1.17, $\chi_{\left(1\right)}^{2}$ = 1.17, *p* = 0.279].

#### Latency

AS latency on correct trials (mean = 440.86, *SD* = 59.62) had a negative relationship with participant age [*t* = −3.32, $\chi_{\left(1\right)}^{2}$ = 11.01, *p* = 0.001]. AS latency was significantly shorter in rewarded trials compared to neutral trials [*t* = −6.99, $\chi_{\left(1\right)}^{2}$ = 48.89, *p* < 0.001].

#### Substance use and risk factors

Of the substance use and risk measures, only positive urgency was significantly associated with AS latency, where higher positive urgency scores were associated with shorter latencies [*t* = −2.80, $\chi_{\left(1\right)}^{2}$ = 7.84, *p* = 0.005] (see Table 4). The relationship between positive urgency and AS latency did not vary by reward condition [*t* = 0.42, $\chi_{\left(1\right)}^{2}$ = 0.18, *p* = 0.676].

#### Covariate relationships

Neither SES [*t* = 0.55, $\chi_{\left(1\right)}^{2}$ = 0.30, *p* = 0.583] nor GA [*t* = 0.24, $\chi_{\left(1\right)}^{2}$ = 0.06, *p* = 0.809] was associated with AS latency. Gender was associated with AS latency, where males (least-squares mean: 421.74 ms) were faster than females (least-squares mean: 457.55 ms) on correct trials [Type A: *t* = −3.55, $\chi_{\left(1\right)}^{2}$ = 12.57, *p* = 0.0003]. When entered as joint predictors (Type A + positive urgency and gender), positive urgency [*t* = −2.47, $\chi_{\left(1\right)}^{2}$ = 6.13, *p* = 0.013] and gender [*t* = −3.22, $\chi_{\left(1\right)}^{2}$ = 10.35, *p* = 0.001] remained significant predictors of AS latency.

### fMRI

#### Head motion

The proportion of censored volumes due to head motion was negatively associated with age [*t* = −2.07, $\chi_{\left(1\right)}^{2}$ = 4.287, *p* = 0.038] and positively associated with both trait impulsivity measures [positive urgency: *t* = 2.86, $\chi_{\left(1\right)}^{2}$ = 8.18, *p* = 0.004; negative urgency: *t* = 2.80 $\chi_{\left(1\right)}^{2}$ = 7.86, *p* = 0.005]. There was also trend for those in the externalizing risk group to have more motion [*t* = 1.94 $\chi_{\left(1\right)}^{2}$ = 3.77, *p* = 0.052]. In contrast, the proportion of censored volumes due to motion was not associated with exceeding threshold drinking [*t* = 0.35, $\chi_{\left(1\right)}^{2}$ = 0.12, *p* = 0.728], internalizing risk [*t* = −1.16, $\chi_{\left(1\right)}^{2}$ = 1.35, *p* = 0.244], or family history of SUD [*t* = 0.50, $\chi_{\left(1\right)}^{2}$ = 0.25 *p* = 0.617]. As detailed in the method section, the proportion of censored volumes due to head motion was used as a covariate in all fMRI analyses.

#### Task effects

**Figure 3:**
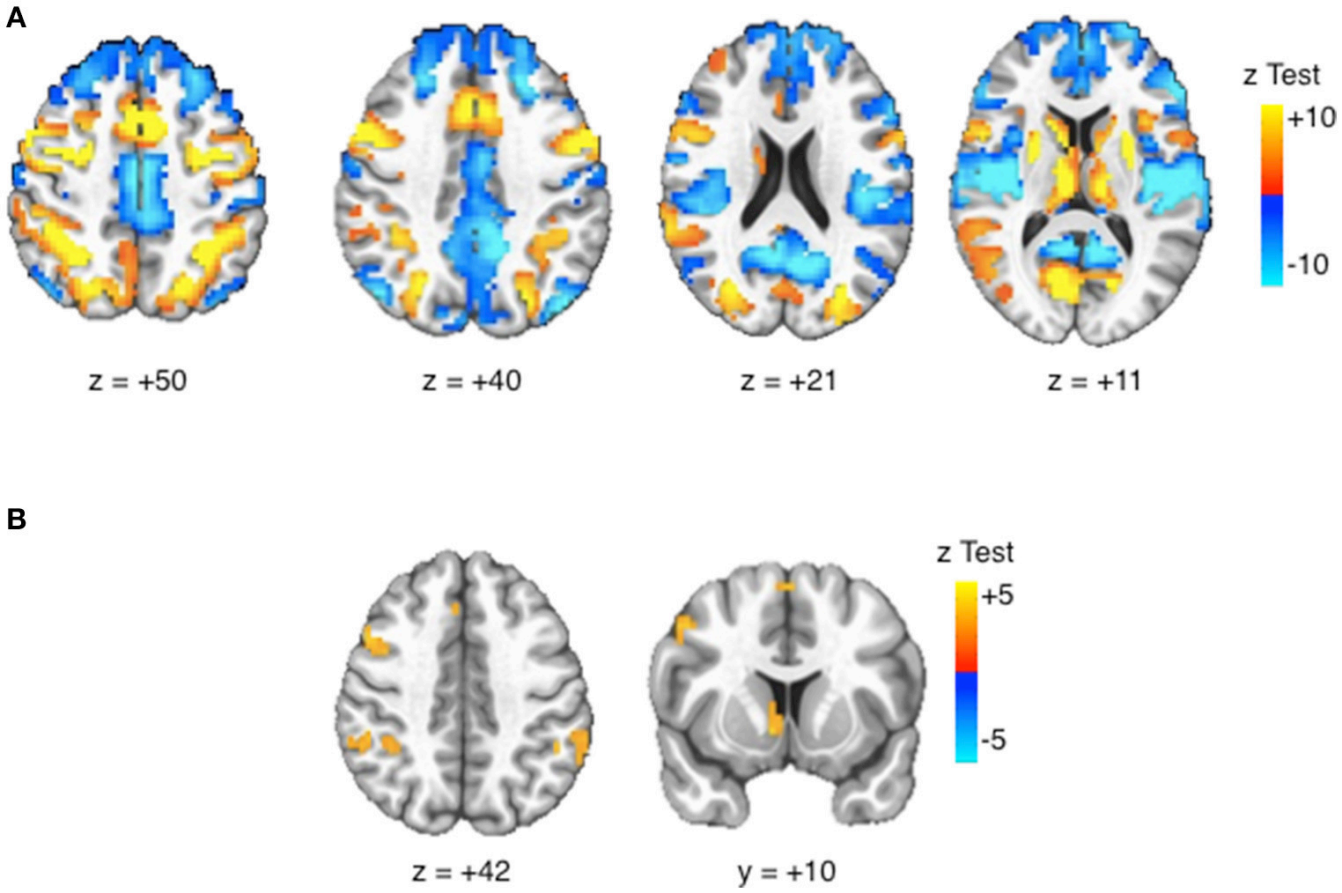
Task BOLD effects. Activation maps displayed at voxelwise threshold *p* < 0.005, number of contiguous voxels (faces touching) >24, cluster-level alpha <0.05. **(A)** Task > Fixation: Robust BOLD activation is observed in canonical eye movement and executive function areas. **(B)** Reward > Neutral: Increased BOLD activation is observed in striatal reward areas and attentions areas.

Robust activation in response to the task (task > fixation) was observed in canonical AS and cognitive control regions, including bilateral frontal eye fields (FEF), posterior parietal cortices (PPC), as well as in the anterior cingulate cortex (ACC) and striatum (see Figure 3). All selected ROIs had significant positive BOLD activation (task > fixation) in either the neutral or reward condition (Table 3). Increased activation to reward was observed at the voxelwise level in the striatum, bilateral PPC, and the right middle frontal gyrus/dorsolateral prefrontal cortex (DLPFC; Figure 3). The majority of ROIs had greater activation in reward trials, compared to neutral (Table 3).

#### Main effects of ETD and substance use risk factors

Within a priori ROIs, positive urgency was associated with reduced BOLD activation in the putamen [*t* = −4.68, $\chi_{\left(1\right)}^{2}$ = 21.86, *p* < 0.001, corrected], FEF [*t* = −2.64, $\chi_{\left(1\right)}^{2}$ = 6.96, *p* = 0.031, corrected], and inferior frontal gyrus [IFG; *t* = −3.00, $\chi_{\left(1\right)}^{2}$ = 8.99, *p* = 0.015, corrected] (Table 5). Family history of SUD was associated with reduced activation in the posterior parietal cortex, although this was only a trend after multiple comparison correction [*t* = −2.74, $\chi_{\left(1\right)}^{2}$ = 7.49, *p* = 0.006 uncorrected, *p* = 0.068 corrected]. These effects were unchanged with ETD and all substance use risk measures entered in a multivariate model (Type B) or when including SES and GA as covariates (Type C). Furthermore, the association between positive urgency and BOLD activation in the putamen, FEF, and IFG, remained significant while covarying gender. No other measures had significant or trending, corrected main effects.

**Table 5:** BOLD main effects in regions of interest (*t*-values): trial-wise (GLM-1).

	**EXT**	**INT**	**FH**	**ETD**	**PUG**	**NUG**	**Age**	**SES**	**GA**	**AS Acc**
**SUBCORTICAL**										
Caudate^L.R.^	−1.36	−0.41	−1.03	0.04	−1.00	1.55	−1.65	−0.47	0.41	2.48^*^
Putamen^L.R.^	−**2.29**	−1.24	−1.33	−0.19	−4.68^*^^B^^,^^C^^,^^G^	−1.35	0.87	−0.30	0.44	3.08^*^
NAcc^L.R.^	−1.38	−0.21	0.06	0.45	1.39	1.68	−0.86	0.70	0.81	−0.14
**CORTICAL**										
PPC^L.R.^	−0.91	−0.33	−**2.74**^+^^B^^,^^C^	−**2.42**	−0.51	−0.20	0.10	−0.58	0.64	1.72
FEF^L.R.^	−**2.05**	−0.79	−0.78	0.20	−2.64^*^^B^^,^^C^^,^^G^	−0.87	1.03	0.23	**2.09**	**2.34**^+^
SEF	−1.45	−0.63	−0.23	0.89	−1.06	−0.75	−0.34	−0.77	−0.55	**2.04**^+^
Pre-SMA	−1.79	0.25	−0.26	−1.22	−1.70	−1.88	0.34	0.06	0.84	**2.14**^+^
dACC	−1.53	−0.54	−0.88	−0.55	−1.59	−0.93	−0.94	−0.30	1.11	2.57^*^
DLPFC^L.R.^	−0.36	−0.10	−1.51	−1.25	−1.65	−0.12	−1.34	−0.62	−1.46	0.49
VLPFC^L.R.^	−0.90	−1.31	−1.04	0.07	−1.34	−0.64	1.35	−1.28	0.47	−1.21
IFG^L.R.^	0.43	−0.48	−1.47	−0.80	−3.00^*^^B^^,^^C^^,^^G^	−0.26	−0.05	−1.03	−1.31	1.16

+ *p < 0.10 (corrected)*,

* *p < 0.05 (corrected). Displayed estimates are test statistics from models with the specific factor, subject age, visit, and reward condition (Type A)*.

B p < 0.05 (Type B) model with all risk factors, subject age, visit, and reward condition;

C *p < 0.05 (Type C) model with all risk factors, subject age, visit, reward condition, and SES and GA*.

G *Positive Urgency p < 0.05 while covarying gender*.

L.R. *, Left/Right ROIs are included within one model. Estimates with uncorrected p's < 0.05 are bolded*.

**Figure 4:**
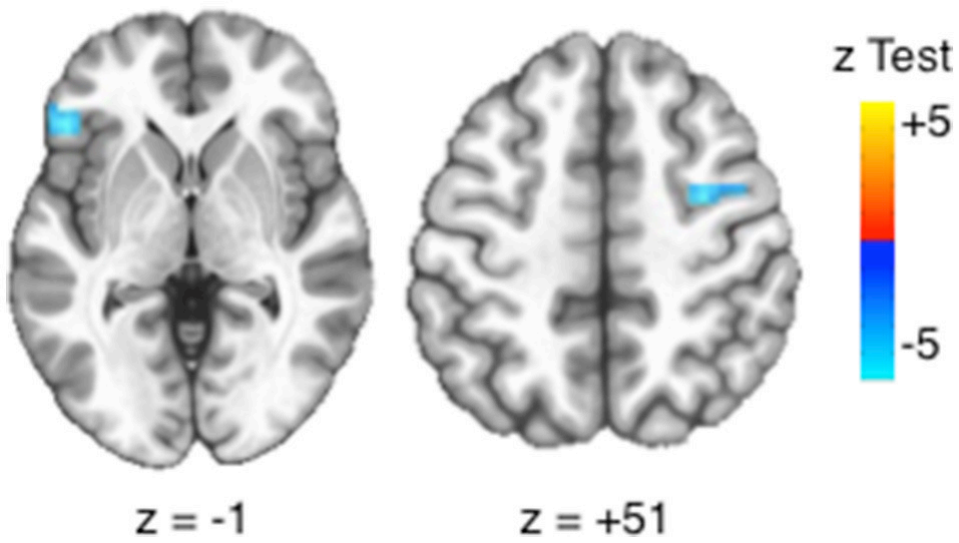
Voxelwise main effects of positive urgency. Positive urgency had a significant, voxelwise main effect with BOLD activation in the left inferior frontal gyrus (IFG: *x* = 49.5, *y* = 28.5, *z* = −1.5; 32 voxels; peak test statistic = −5.93) and right frontal eye field (FEF; *x* = −31.5, *y* = 1.5, *z* = 49.5, 25 voxels, peak test statistic = −5.52). Significance based on intersection of FDR-correction and cluster size [voxelwise threshold (p) = 0.0012, *q* < 0.05, number of contiguous voxels (faces touching) > 24].

At the voxelwise level, positive urgency had a negative relationship with BOLD activation in the left inferior frontal gyrus (IFG) and right frontal eye field [FEF; voxelwise threshold (*p*) = 0.0012, *q* < 0.05, number of contiguous voxels (faces touching) > 24] (Figure 4), confirming results from a priori ROIs. Negative urgency had two voxels that reached FDR corrected significance (*q* < 0.05). EXT, INT, FH, ETD, and negative urgency did not have significant voxelwise BOLD main effects (minimum *q*'s > 0.072).

### Reward interactions of substance use and risk factors

#### Trial-wise reward interactions

With trial-wise BOLD responses (GLM-1), ETD and substance use risk measures did not have a significant, corrected interaction with reward in a priori ROIs (Table 6) or at the voxelwise level (minimum *q*'s > 0.876).

**Table 6:** BOLD reward interactions in regions of interest (*t*-values): trial-wise (GLM-1).

	**EXT**	**INT**	**FH**	**ETD**	**PUG**	**NUG**	**Age**	**SES**	**GA**	**AS Acc**
**SUBCORTICAL**										
Caudate^L.R.^	−1.12	1.01	0.85	−0.54	0.27	−1.36	0.31	−1.39	−0.28	−1.11
Putamen^L.R.^	0.47	1.75	−0.05	−0.40	−0.11	−0.88	0.28	−1.77	0.30	−0.44
NAcc^L.R.^	−0.21	0.48	0.91	−0.10	−0.27	−1.42	0.09	−1.39	−0.02	0.58
**CORTICAL**										
PPC^L.R.^	0.29	1.26	0.56	0.15	0.84	0.12	−0.11	−0.66	−0.53	−0.03
FEF^L.R.^	−0.21	0.79	0.97	−0.43	1.31	0.18	−0.35	−0.76	0.06	−0.28
SEF	−0.55	0.31	1.73	−0.32	**2.11**	0.16	−0.54	−1.02	−0.30	−0.53
Pre-SMA	−0.64	0.71	1.17	−0.62	0.76	−0.35	−0.15	−0.74	−0.21	−0.43
dACC	−0.71	0.84	0.20	−0.05	0.60	−0.77	0.04	−0.82	−0.26	−0.45
DLPFC^L.R.^	−0.68	1.08	0.97	−0.45	0.35	−1.33	−0.22	−0.82	−0.45	−1.37
VLPFC^L.R.^	−0.21	1.14	−0.10	−0.50	−0.74	−1.74	0.07	−0.58	−0.16	−0.70
IFG^L.R.^	−0.35	1.92	−0.56	0.01	−0.24	−1.13	0.30	−1.13	−0.64	−0.64

*Displayed estimates are test statistics from models with the specific factor, subject age, visit, and reward condition (Type A)*.

L.R. *, Left/Right ROIs are included within one model. Estimates with uncorrected p's < 0.05 are bolded*.

#### Epoch reward interactions

**Figure 5:**
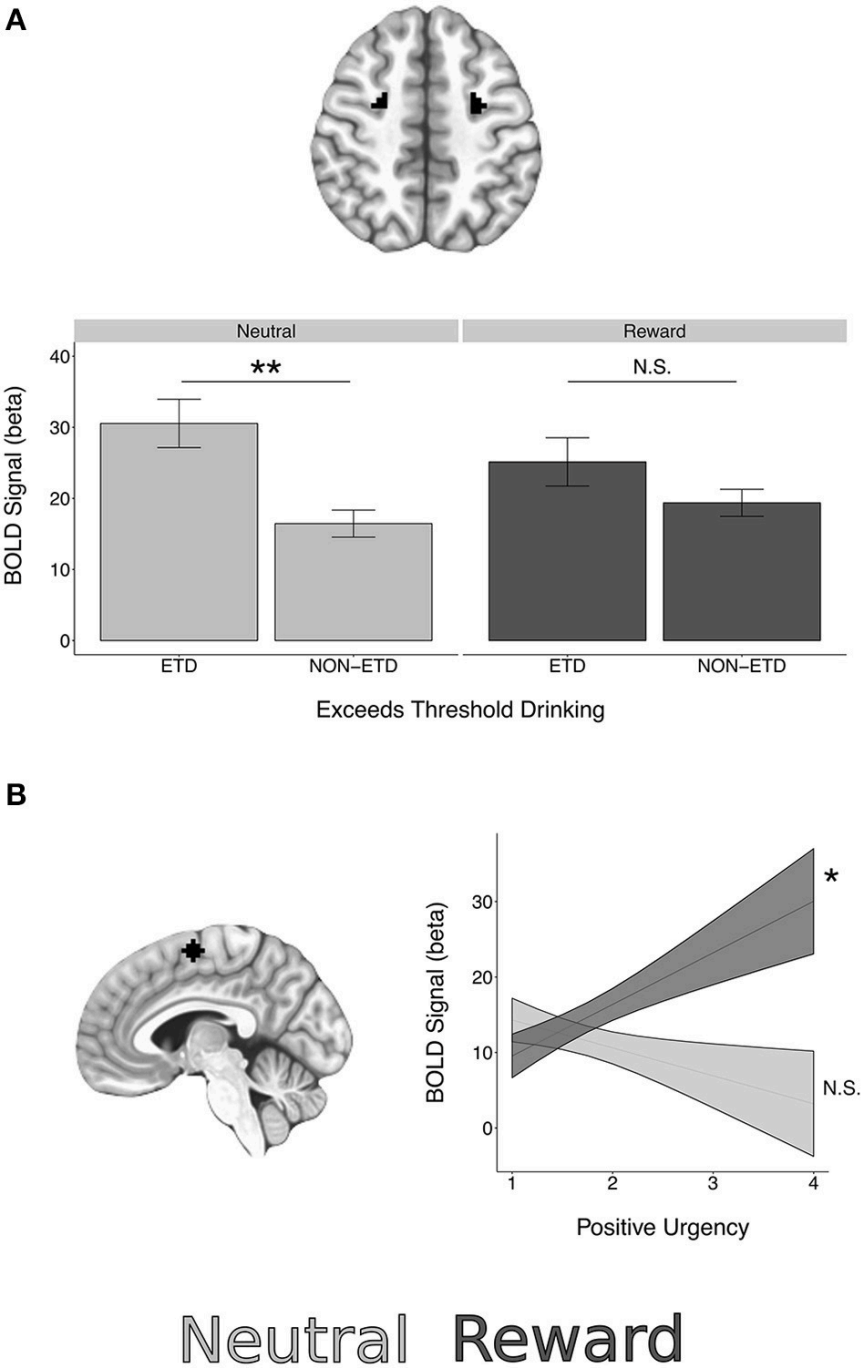
BOLD epoch reward interactions in regions of interest. **(A)** A significant interaction between reward and exceeds threshold drinking was observed in the FEF ROIs during the preparation epoch. **(B)** A significant interaction between reward and positive urgency was observed in the SEF ROI during the response epoch. See Table 7 for interaction statistics and simple effects testing. ^*^p < 0.05, ^**^p < 0.01.

Significant BOLD reward interactions for AS task epochs in ROIs are presented in Table 7. See Supplemental Tables 2–4 for full list. No reward interactions were observed in the cue epoch. In the preparation epoch, there was a significant interaction between subject age and reward condition in the NAcc ROI [*t* = 2.85, $\chi_{\left(1\right)}^{2}$ = 8.10, *p* = 0.048, corrected]. *Post hoc* testing revealed a significant simple effect of age in reward trials [*t*_(204.03)_ = 2.38, *p* = 0.018] but a non-significant simple effect of age in neutral trials [*t*_(204.03)_ = −0.45, *p* = 0.651]. A significant reward interaction was also observed in the preparation epoch for exceeds threshold drinking in the FEF ROI [*t* = −2.92, $\chi_{\left(1\right)}^{2}$ = 8.54, *p* = 0.038, corrected] with *post-hoc* testing demonstrating that those who exceeded threshold drinking had higher BOLD activation than those not meeting criteria in the neutral trials [*t*_(150.98)_ = 3.47, *p* < 0.001], but not reward trials [*t*_(150.98)_ = 1.41, *p* = 0.158] (Figure 5).

**Table 7:** BOLD reward interactions of antisaccade epochs in regions of interest (GLM-2).

	**Variable**	**Reward interaction (*t*)**	**Simple effect: neutral (*t*)**	**Simple effect: reward (*t*)**
**PREPARATION**				
NAcc^L.R.^	Age	2.85^*^^B^^,^^C^	−0.45	2.38^*^
FEF^L.R.^	ETD	−2.92^*^^B^^,^^C^	3.47^**^	1.41
**RESPONSE**				
SEF	PUG	3.06^*^^B^^,^^C^^,^^G^	−1.10	2.24^*^
IFG^L.R.^	EXT	2.96^*^^B^^,^^C^	−1.65	0.93

*Reward interaction estimates are test statistics from models with the specific variable, subject age, visit, and reward condition (Type A) that were significant after multiple comparison correction. See Supplemental Tables 2–4 for full epoch reward interactions*.

B p < 0.05 (Type B) model with all risk factors, subject age, visit, and reward condition;

C *p < 0.05 (Type C) model with all risk factors, subject age, visit, reward condition, and SES and GA*.

G *Positive Urgency p < 0.05 while covarying gender*.

L.R. *, Left/Right ROIs are included within one model*.

Simple effect neutral and reward refer to test statistics from association between variable and BOLD activation in neutral and reward trials, respectively:

* *p < 0.05*,

** *p < 0.01*.

In the response epoch, there was a significant reward interaction for positive urgency in the SEF [*t* = 3.06, $\chi_{\left(1\right)}^{2}$ = 9.35, *p* = 0.024, corrected], with *post-hoc* testing demonstrating a significant simple effect of positive urgency in reward trials [*t*_(323.86)_ = 2.24, *p* = 0.026] but a non-significant simple effect in neutral trials [*t*_(323.86)_ = −1.10, *p* = 0.274] (Figure 5). A reward interaction was also observed for externalizing risk in the IFG during the response epoch [*t* = 2.96, $\chi_{\left(1\right)}^{2}$ = 8.75, *p* = 0.034, corrected], but *post-hoc* testing revealed non-significant simple effects in both reward and neutral trials (*p*'s > 0.0998). However, no reward interactions were significant at the voxelwise level (minimum *q*'s > 0.107).

## Discussion

In this study, we examined neural correlates of rewarded response inhibition in youth at risk for problematic substance use. Within a two time point adolescent neuroimaging dataset, we tested whether incentives moderated the association between response inhibition and trait-level psychopathology and personality features of substance use risk. In multivariate models, the trait impulsivity measure of positive urgency and externalizing risk were significant negative predictors of AS correct response rate. Positive urgency was associated with reduced trial-wise BOLD activation in a priori ROIs representing frontal eye fields and inferior frontal gyrus, which were confirmed at the voxelwise level. However, contrary to our hypothesis, limited evidence suggested differential associations between response inhibition and substance use risk factors in the context of reward. Significant interactions between reward and substance use risk factors were not observed with AS behavioral performance or trial-wise BOLD estimates. When examining reward effects in discrete stages of the AS task (e.g., cue, preparation, response), some reward interactions were observed in a priori ROIs, but no reward interaction effects were observed at the voxelwise level.

### Substance use risk and brain systems supporting response inhibition

Consistent with previous work (Young et al., 2009), externalizing risk was associated with poorer response inhibition. However, externalizing risk was also associated with lower general cognitive ability (GA) and when GA was included as a covariate, the association between externalizing risk and AS performance was reduced to a trend. This suggests the association between externalizing risk and AS performance may be driven in part, by aspects of cognition that are not specific to inhibition. In contrast, higher levels of the trait impulsivity measure of positive urgency were associated with poorer AS performance, while accounting for GA. Higher scores of positive urgency also predicted shorter latencies to correct AS responses. This pattern is consistent with the notion of high levels of trait impulsivity predicting a speed-accuracy tradeoff in cognitive tests (Dickman and Meyer, 1988). We further demonstrate positive urgency is associated with reduced BOLD activation in the inferior frontal gyrus (IFG), frontal eye fields (FEF), and putamen. The negative association between urgency and BOLD activation in the IFG is consistent with recent work examining UPPS-P urgency domain score in a rewarded go/no-go task (Wilbertz et al., 2014). We extend this result by demonstrating this association may be specific to positive urgency, rather than negative urgency.

Positive urgency is conceptualized as the tendency to act rashly/impulsively in response to high levels of positive affect (Cyders et al., 2007). Theoretical models implicate the ventromedial prefrontal cortex (VMPFC) in affective instability and impulsivity (see Cyders and Smith, 2008), highlighting evidence from patients with damage to this region (Bechara, 2004). However, in the absence of a significant interaction between positive urgency and reward in AS behavioral performance, our results suggest positive urgency is associated with a general response inhibition deficit. Accordingly, we observed negative associations between positive urgency and BOLD activation in multiple regions associated with response inhibition, including IFG (c.f., Aron et al., 2003) and FEF (c.f., Muggleton et al., 2010) and the putamen ROI (Zandbelt and Vink, 2010).

Both positive urgency and externalizing risk were significant negative predictors of AS correct response rate. This is consistent with an association between disinhibited psychopathology and poorer response inhibition (Young et al., 2009). Moreover, although not significant after correction for multiple comparisons, externalizing risk was associated with lower BOLD activation in a priori FEF and putamen ROIs. To this end, our data provide some support for a notion of common neurocognitive correlates to disinhibited psychopathology and substance use risk. Moreover, associations between positive urgency and externalizing risk and AS response inhibition appear functionally distinct from other forms of psychopathology, as no other risk factors had significant (corrected or uncorrected) associations with AS behavioral performance or BOLD activation in FEF or the putamen.

### Reward interactions and substance use risk

Consistent with previous work utilizing this rewarded AS task (Geier et al., 2010) and other response inhibition tasks (Hardin et al., 2007), the availability of reward improved response inhibition performance (increased correct response rate and reduced latency). However, no substance use risk measures had significant interactions with reward in AS behavioral performance or trial-wise BOLD estimates. This result may suggest that response inhibition deficits and reward sensitivity may be non-overlapping features of substance use risk, which is consistent with factor analytic work suggesting substance use risk is a multidimensional construct (Woicik et al., 2009).

Previous work suggests BOLD activation associated with reward modulation may vary according to the cognitive processes engaged during different epochs of the AS task (c.f., Geier et al., 2010; Chung et al., 2011). To this end, we observed significant BOLD reward modulation when examining specific AS epochs in a priori ROIs, with exceeding threshold drinking associated with increased BOLD activation in FEF during response preparation in neutral trials, but not reward trials. This result is consistent with previous work using this task in adolescents with SUD (Chung et al., 2011) and provides some support for the notion that incentives may normalize response inhibition differences in SUD. Given the pattern of greater differences in neutral trials compared to reward trials was specific to SUD and not substance use risk factors, its possible this effect reflects a sensitization to rewards following substance use initiation. To this end, positive urgency was associated with increased BOLD activation during response preparation in the supplementary eye fields during reward trials, but not neutral trials. However, reward interactions were not observed at the voxelwise level, indicating the need for caution in interpreting possible effects of reward on regional BOLD activation.

A possible explanation for a lack of reward modulation of the association between response inhibition and substance use risk factors in this study may be from our focus on general associations across development. Previous work suggests developmental changes within reward circuitry during adolescence (Larsen and Luna, 2015). Supporting this we found age by reward interactions in the NAcc during the preparation epoch, with greater age-related changes in reward trials compared to neutral. This result is consistent with previous work (Bjork et al., 2010b) suggesting adults have greater NAcc activation during reward anticipation. Accordingly, reward interactions with substance use risk may emerge later in development (Müller et al., 2015). The degree of the moderating effect of reward on the association between substance use risk factors and response inhibition may also depend on the magnitude of the incentive offered. Given the ongoing data-collection within the NCANDA sample, future work may explore within-subject, longitudinal changes during development in relation to reward interactions with substance use risk.

### Limitations

This project was characterized by a number of strengths, including a relatively large sample size, multiple time points (baseline and follow up visits), and extensive characterization of psychopathology and personality variables associated with substance use risk. However, it is worth noting a few limitations. First, we coded participants risk scores at both baseline and follow-up visits based on baseline risk factors established by NCANDA. Combined with the use of mixed effects models, this procedure allowed us to increase the precision of between-subject estimates of risk factors groups as defined in previous NCANDA projects (c.f., Brown et al., 2015) and utilize all data from baseline and follow up visits. Nevertheless, subtle variation in meeting criteria for certain risk factors (e.g., externalizing and internalizing risk and exceeding threshold drinking) may have occurred between baseline and follow up visits and we did not examine within-subject change (difference scores between baseline and 1-year follow-up). Accordingly, future work may utilize more complex modeling frameworks with random growth terms to better characterize within-subject changes and joint maturation of substance use risk and brain activation.

Another potential limitation of the analyses is the focus on bivariate relationships between substance use risk factors and AS performance and BOLD activation. Although we examined interactions between substance use risk and reward processes, these were completed iteratively across risk factors. This procedure, combined with multivariate regression, allowed us to examine the specificity of associations between particular substance use risk factors and AS performance and BOLD activation. However, several of our risk factors were significantly correlated with one another. To this end, previous work suggests substance use risk may be characterized by higher-order, latent dimensions that may explain risk factor covariation (c.f., Tarter et al., 2003; Woicik et al., 2009). Future work could utilize latent variable analysis to examine whether higher-order factors of substance use risk display significant interactions with reward on response inhibition tasks.

## Conclusion

Utilizing a rewarded antisaccade task during fMRI acquisition, the results from this project confirm previous work suggesting substance use risk, and specifically externalizing psychopathology and trait impulsivity, are associated with poorer response inhibition. Furthermore, we found that higher levels of positive urgency are associated with reduced BOLD activation in FEF and IFG. However, we found little evidence that monetary incentive moderated the association between substance use risk factors and AS behavioral performance or BOLD activation. Further work is needed to determine the parameters (e.g., type, magnitude of reward) under which incentives increase response inhibition, examine overlap between substance use risk factors, and investigate within-subject longitudinal change in the interplay between sensitivity to reward and response inhibition.

## Author contributions

BT, BL, and DC designed analysis. BT and AQ completed analysis. BT and WF performed preprocessing and quality assurance on imaging data. BT constructed manuscript with input and feedback from BL, TC, MD, and DC.

### Conflict of interest statement

The authors declare that the research was conducted in the absence of any commercial or financial relationships that could be construed as a potential conflict of interest. The reviewer SN and handling Editor declared their shared affiliation.
