# Supplementary material for: Neural Correlates of Rewarded Response Inhibition in Youth at Risk for Problematic Alcohol Use
Source: Front Behav Neurosci. 2017 Nov 3;11:205. doi: 10.3389/fnbeh.2017.00205 (PMC5675888; doi:10.3389/fnbeh.2017.00205)
Supplement: Supplementary file 1 [file Table1.docx]

**Supplemental Table 1. Association between Proportion of Excluded Eye Tracking Trials and Study Variables**

| **EXT** | **INT** | **FH** | **ETD** | **PUG** | **NUG** | **Age** | **SES** | **GA** |
| --- | --- | --- | --- | --- | --- | --- | --- | --- |
| 0.44 | 0.62 | **-1.97*** | -0.06 | 1.14 | **2.01*** | -0.48 | -0.32 | -0.17 |

**Note.** * p < .05. Displayed Estimates are t statics from models predicting the percentage of poor eye tracking trials.
