# Supplementary material for: Neural Correlates of Rewarded Response Inhibition in Youth at Risk for Problematic Alcohol Use
Source: Front Behav Neurosci. 2017 Nov 3;11:205. doi: 10.3389/fnbeh.2017.00205 (PMC5675888; doi:10.3389/fnbeh.2017.00205)
Supplement: Supplementary file 2 [file Table2.DOCX]

**Supplemental Table 2.** BOLD Reward Interactions of the Cue Epoch in Regions of Interest (t-values)

|  | **EXT** | **INT** | | **FH** | **ETD** | **PUG** | **NUG** | **Age** | **SES** | **GA** | **AS Acc** |
| --- | --- | --- | --- | --- | --- | --- | --- | --- | --- | --- | --- |
| **Subcortical** |  |  |  | |  |  |  |  |  |  |  |
| *Caudate* ^L.R.^ | -0.59 | -0.27 | 0.13 | | -0.86 | -0.04 | -2.30 | -1.02 | 0.25 | -0.55 | -1.64 |
| *Putamen* ^L.R.^ | 0.80 | 0.18 | -0.71 | | -0.74 | 1.15 | 0.05 | -0.66 | -0.31 | -1.60 | -1.40 |
| *NAcc* ^L.R.^ | 0.34 | 1.45 | 0.06 | | -0.86 | 1.54 | -1.11 | -2.53 | -1.29 | **-2.76** | -0.30 |
| **Cortical** |  |  |  | |  |  |  |  |  |  |  |
| *PPC* ^L.R.^ | 0.47 | 0.55 | -0.72 | | 0.85 | 0.78 | -0.38 | -0.41 | 0.78 | -1.19 | -1.28 |
| *FEF*  ^L.R.^ | 0.19 | -0.99 | -0.99 | | 1.65 | 0.22 | -1.30 | -0.78 | 1.85 | -1.51 | -1.92 |
| SEF | -1.04 | -1.07 | -1.51 | | 1.16 | -0.35 | -1.34 | -0.09 | 1.54 | -0.33 | -1.17 |
| *Pre-SMA* | -0.86 | -0.36 | -1.04 | | 1.52 | -0.32 | -0.84 | 0.51 | 1.68 | 0.26 | -1.37 |
| dACC | -0.19 | -0.51 | -1.09 | | 0.89 | -0.46 | -0.76 | 0.64 | 0.56 | -0.53 | -0.67 |
| *DLPFC* ^L.R.^ | -0.74 | 0.37 | 0.54 | | 0.21 | -0.35 | -1.86 | -0.14 | 0.71 | 0.21 | -1.47 |
| VLPFC ^L.R.^ | -0.42 | -1.08 | -1.40 | | 0.80 | -0.40 | -1.29 | 0.84 | 0.97 | -0.51 | **-2.16** |
| IFG ^L.R.^ | -0.42 | -0.21 | -1.29 | | -0.49 | -0.12 | -1.37 | -0.47 | -0.55 | -1.86 | -1.44 |

**Note.** Displayed estimates are test statistics from models with the specific factor, subject age, visit, and reward condition (Type A). Estimates with uncorrected p’s < .05 are bolded.
