# Supplementary material for: Neural Correlates of Rewarded Response Inhibition in Youth at Risk for Problematic Alcohol Use
Source: Front Behav Neurosci. 2017 Nov 3;11:205. doi: 10.3389/fnbeh.2017.00205 (PMC5675888; doi:10.3389/fnbeh.2017.00205)
Supplement: Supplementary file 3 [file Table3.DOCX]

|  | **EXT** | **INT** | | **FH** | **ETD** | **PUG** | **NUG** | **Age** | **SES** | **GA** | **AS Acc** |
| --- | --- | --- | --- | --- | --- | --- | --- | --- | --- | --- | --- |
| **Subcortical** |  |  |  | |  |  |  |  |  |  |  |
| *Caudate* ^L.R.^ | -0.69 | 0.76 | 0.47 | | -0.98 | -0.43 | 0.29 | 0.35 | -0.36 | -1.19 | 0.76 |
| *Putamen* ^L.R.^ | -1.92 | -0.19 | 0.77 | | 0.41 | -1.39 | -0.68 | 0.29 | -0.53 | 0.44 | **2.23*** |
| *NAcc* ^L.R.^ | 0.94 | -1.08 | 0.69 | | 0.17 | -1.42 | 0.29 | **2.85*** | 0.27 | 1.36 | 0.57 |
| **Cortical** |  |  |  | |  |  |  |  |  |  |  |
| *PPC* ^L.R.^ | -1.13 | 0.97 | 0.91 | | -0.51 | -0.81 | -0.08 | 1.73 | -0.98 | 1.00 | **2.82*** |
| *FEF*  ^L.R.^ | -0.66 | 1.06 | 0.00 | | **-2.92*** | -0.67 | -0.34 | 0.25 | **-2.09** | 0.30 | **3.24*** |
| SEF | 0.11 | 1.29 | 1.23 | | -1.92 | -0.60 | 0.17 | 0.64 | **-2.18** | 0.14 | **2.46*** |
| *Pre-SMA* | -0.06 | 0.52 | 0.84 | | -1.89 | -0.66 | -0.38 | -0.10 | -1.86 | -0.42 | **2.56*** |
| dACC | -0.71 | 0.90 | 0.11 | | -1.31 | 0.05 | -0.49 | -0.09 | -1.06 | 0.35 | 1.20 |
| *DLPFC* ^L.R.^ | -0.15 | 0.74 | -0.06 | | 0.41 | -0.01 | 0.47 | 1.73 | -0.51 | -0.24 | 1.52 |
| VLPFC ^L.R.^ | -0.99 | 1.89 | 0.05 | | -1.24 | 0.15 | 0.45 | -1.63 | -0.32 | -0.10 | **3.09*** |
| IFG ^L.R.^ | **-2.05** | 0.79 | 0.74 | | -0.37 | 0.06 | 0.42 | -0.48 | 0.83 | 1.62 | 1.64 |

**Supplemental Table 3.** BOLD Reward Interactions of the Preparation Epoch in Regions of Interest (t-values)

**Note.** Displayed estimates are test statistics from models with the specific factor, subject age, visit, and reward condition (Type A). Estimates with uncorrected p’s < .05 are bolded
