# Supplementary material for: Neural Correlates of Rewarded Response Inhibition in Youth at Risk for Problematic Alcohol Use
Source: Front Behav Neurosci. 2017 Nov 3;11:205. doi: 10.3389/fnbeh.2017.00205 (PMC5675888; doi:10.3389/fnbeh.2017.00205)
Supplement: Supplementary file 4 [file Table4.DOCX]

**Supplemental Table 4.** BOLD Reward Interactions of the Response Epoch in Regions of Interest (t-values)

|  | **EXT** | **INT** | | **FH** | **ETD** | **PUG** | **NUG** | **Age** | **SES** | **GA** | **AS Acc** |
| --- | --- | --- | --- | --- | --- | --- | --- | --- | --- | --- | --- |
| **Subcortical** |  |  |  | |  |  |  |  |  |  |  |
| *Caudate* ^L.R.^ | 0.35 | 0.30 | 0.33 | | 1.36 | 1.21 | 0.37 | 0.75 | -1.09 | 1.93 | -0.56 |
| *Putamen* ^L.R.^ | **2.50+** | 1.53 | -0.44 | | -0.54 | 1.48 | 0.63 | 0.17 | -0.53 | 0.51 | -1.74 |
| *NAcc* ^L.R.^ | -1.34 | 0.97 | -0.25 | | 0.23 | 0.70 | -0.50 | -1.82 | -0.71 | 0.12 | -0.33 |
| **Cortical** |  |  |  | |  |  |  |  |  |  |  |
| *PPC* ^L.R.^ | 1.67 | 0.13 | -0.11 | | 0.18 | 1.99 | 0.92 | -1.75 | -0.29 | -0.97 | -1.92 |
| *FEF*  ^L.R.^ | 0.71 | 0.43 | 1.36 | | **2.10** | **2.49+** | 1.50 | 0.01 | 0.63 | 0.68 | **-2.35+** |
| SEF | 0.30 | -0.46 | 0.82 | | 1.43 | **3.06*** | 1.06 | -1.13 | 0.98 | 0.05 | **-2.57+** |
| *Pre-SMA* | 0.55 | 0.39 | 0.50 | | 0.76 | **2.26** | 1.14 | -0.30 | 0.45 | 0.17 | **-2.34+** |
| dACC | 0.72 | -0.12 | 0.54 | | 1.06 | 1.18 | 0.80 | 0.02 | 0.11 | -0.25 | -1.15 |
| *DLPFC* ^L.R.^ | 0.29 | -0.16 | 0.33 | | -1.07 | 1.25 | 0.05 | -2.01 | -0.91 | -0.47 | -1.91 |
| VLPFC ^L.R.^ | 1.61 | -0.09 | 0.73 | | 0.12 | 0.00 | -0.96 | 1.49 | -1.38 | 0.33 | **-2.46+** |
| IFG ^L.R.^ | **2.96*** | 0.72 | -0.66 | | 0.67 | 0.14 | -0.44 | 1.28 | -1.94 | -1.54 | -1.05 |

**Note.** Displayed estimates are test statistics from models with the specific factor, subject age, visit, and reward condition (Type A). Estimates with uncorrected p’s < .05 are bolded.
